# Improving the clinical interpretation of missense variants in X linked genes using structural analysis

**DOI:** 10.1136/jmedgenet-2020-107404

**Published:** 2021-03-25

**Authors:** Shalaw Rassul Sallah, Jamie M Ellingford, Panagiotis I Sergouniotis, Simon C Ramsden, Nicholas Lench, Simon C Lovell, Graeme C Black

**Affiliations:** 1 Division of Evolution and Genomic Sciences, The University of Manchester Faculty of Biology, Medicine and Health, Manchester, UK; 2 Manchester Centre for Genomic Medicine, St Mary’s Hospital, Manchester Academic Health Sciences Centre, Manchester, UK; 3 Congenica Ltd, Biodata Innovation Centre, Wellcome Genome Campus, Hinxton, London, UK

**Keywords:** clinical decision-making, genetic variation, mutation, missense, structural homology, protein, point mutation

## Abstract

**Background:**

Improving the clinical interpretation of missense variants can increase the diagnostic yield of genomic testing and lead to personalised management strategies. Currently, due to the imprecision of bioinformatic tools that aim to predict variant pathogenicity, their role in clinical guidelines remains limited. There is a clear need for more accurate prediction algorithms and this study aims to improve performance by harnessing structural biology insights. The focus of this work is missense variants in a subset of genes associated with X linked disorders.

**Methods:**

We have developed a protein-specific variant interpreter (ProSper) that combines genetic and protein structural data. This algorithm predicts missense variant pathogenicity by applying machine learning approaches to the sequence and structural characteristics of variants.

**Results:**

ProSper outperformed seven previously described tools, including meta-predictors, in correctly evaluating whether or not variants are pathogenic; this was the case for 11 of the 21 genes associated with X linked disorders that met the inclusion criteria for this study. We also determined gene-specific pathogenicity thresholds that improved the performance of VEST4, REVEL and ClinPred, the three best-performing tools out of the seven that were evaluated; this was the case in 11, 11 and 12 different genes, respectively.

**Conclusion:**

ProSper can form the basis of a molecule-specific prediction tool that can be implemented into diagnostic strategies. It can allow the accurate prioritisation of missense variants associated with X linked disorders, aiding precise and timely diagnosis. In addition, we demonstrate that gene-specific pathogenicity thresholds for a range of missense prioritisation tools can lead to an increase in prediction accuracy.

## Introduction

Advances in high-throughput DNA sequencing technologies have transformed how clinical diagnoses are made in individuals and families with Mendelian disorders. Genetics tests using these approaches are now widely used in the clinical setting, reducing diagnostic uncertainty and improving patient management.^1^ Notably, results of these tests are often ambiguous and it is common for these investigations to yield a number of variants of uncertain significance (VUS). Interpreting these VUS is not a trivial task and numerous in silico prediction tools have been developed to filter and prioritise such changes for further analysis. However, these tools lack robustness and are commonly inconsistent in their predictions^2 3^ and their performance.^4^ Taking this into account, the American College of Medical Genetics and Genomics (ACMG) and the Association for Molecular Pathology guidelines for variant interpretation^5^ have concluded that bioinformatics tools can provide only supporting evidence for pathogenicity. Improving the performance of these algorithms is expected to have significant implications for variant interpretation and ultimately for clinical decision making.

In a previous study, we integrated genetic and structural biology data to predict variant–disease association with high accuracy in the X linked gene *CACNA1F* (MIM: 300110); the area under the receiver operating characteristic (ROC) and precision–recall (PR) curves was 0.84; Matthews correlation coefficient (MCC) was 0.52.^6^ Here, we replicate the accuracy and robustness of this approach in several other disease-implicated X linked genes. Furthermore, we evaluate seven prediction tools and show that the meta-predictors REVEL (rare exome variant ensemble learner),^7^ VEST4 (variant effect scoring tool 4.0),^8^ and ClinPred^9^ are generally the most accurate in predicting the impact of missense variants in this group of disorders. We also show that applying a gene-specific pathogenicity threshold when using these tools can improve their performance at least for some genes. More importantly, we demonstrate that the protein-specific variant interpreter (ProSper) that we developed as part of this study performs better than REVEL, VEST4 and ClinPred in 11 of the 21 studied genes. These insights can help clinicians and diagnostic laboratories better prioritise missense changes in these molecules.

## Methods

### Missense variant data sets

The Human Gene Mutation Database (HGMD V.2019.4)^10^ was used to retrieve missense variants that have been associated with disease (marked ‘DM’), that is, presumably pathogenic. The Genome Aggregation Database (gnomAD V.2.1.1)^11^ was used to retrieve benign/likely benign missense variants reported in males. The variants present in gnomAD which were also present in HGMD as ‘DM?’, that is, disease association is dubious, or as ‘DM’ were filtered out to minimise the inclusion of possible misannotated variants. Missense changes reported in patients tested at the Manchester Genomic Diagnostic Laboratory (MGDL), a UK accredited genomic diagnostic laboratory (Clinical Pathology Accredited identifier no 4015), were also included; these were classified using the ACMG guidelines. The rare as well as the common variants reported in gnomAD were included in order for the model to differentiate the benign rare variants from the pathogenic changes.^7^ We limited our analyses to X linked genes from HGMD that contained a minimum of 70 pathogenic missense variants as informed by earlier findings.^6^

### Protein structures and homology modelling

Experimentally determined three-dimensional (3D) structures were used to perform structural analysis, where available. Otherwise, a homologous model of the protein was generated using either SWISS-MODEL^12^ or RaptorX.^13^ These resources provide the results of alignments and sequence identity of multiple templates informing model selection; RaptorX also allows the production of multi-template models. The protein sequences of the transcript used in HGMD were obtained from UniProt database.^14^ PyMOL^15^ was used to visualise the structures/models.

### Performance assessment of *in silico* tools

A number of prediction tools were evaluated using the two variant data sets assembled above. These included SIFT (Sorting Intolerant From Tolerant),^16^ which uses sequence homology or conservation, and PolyPhen2 (Polymorphism Phenotyping v2),^17^ which uses sequence homology combined with structural properties.^18^ Other tools assessed in this study included the later generation meta-predictors REVEL,^7^ VEST4,^8^ ReVe (a combination of the predictions of REVEL and VEST4),^19^ ClinPred^9^ and CAPICE (Consequence-Agnostic prediction of Pathogenicity Interpretation of Clinical Exome variations).^20^ Most meta-predictors use multiple features in addition to the predictions of algorithms like SIFT and PolyPhen2. ClinPred and CAPICE were shown to perform well on different data sets.^9 20^ The variants’ prediction scores for SIFT, PolyPhen2, REVEL and VEST4 were obtained from the non-synonymous functional prediction database V.4 (dbNSFP^21^). ReVe,^19^ ClinPred^9^ and CAPICE^20^ prediction scores were obtained from their respective databases. The pathogenicity thresholds used for SIFT, PolyPhen2, VEST4, REVEL, ReVe, ClinPred and CAPICE were 0.05, 0.85, 0.5, 0.5, 0.7, 0.5 and 0.02, respectively, as suggested in the relevant specifications. The performance of the tools was measured through the area under the curve (AUC) of the ROC^22^ and the PR^23^ curves. MCC^24^ was used to measure the correlation between the observed variant class and the predictions made by the tools.

### Variant features and analyses

As in our previously described methodology,^6^ a set of sequence-based and structure-based features were defined to integrate clinical and structural data. The ‘colour’ column obtained from ConSurf server,^25^ using default parameters, was used to measure conservation at the variant site instead of using a protein-specific multiple sequence alignment. Differences in residue volume were calculated using the Richards scale.^26^ Changes in hydrophobicity were identified using a previously described hydrophobicity scale which identifies seven residues as strongly hydrophobic.^27^ In addition to these, a number of other features were considered. Side-chain solvent accessibility was measured using Naccess.^28^ Information on functional sites, protein and topological domains, and secondary structure was directly obtained from the UniProt database, when available. The predicted secondary structure of the protein was otherwise obtained using PyMOL. The probability of a variant affecting protein stability or protein–protein interaction was measured using predictions made by mCSM (mutation Cutoff Scanning Matrix).^29^ Variants predicted to be in intrinsically disordered regions of the protein were identified using IUPRED2A (an algorithm for predicting intrinsically unstructured/disordered proteins and domains).^30^ Variants involving residues with special physicochemical characteristics were predicted to affect protein structure and function, for example, the introduction of proline onto β-strands, the introduction/loss of glycine in the core or the introduction/loss of cysteine in extracellular regions possibly leading to the breakage of disulfide bridges. The features which could be retrieved and used for variant annotation in all genes were named ‘general features’; these included physicochemical changes, solvent accessibility, molecular goodness-of-fit and conservation. Other features which could only be retrieved and used for variant annotation in certain genes were named ‘gene-specific features’; these included variant clustering and protein information such as functional domains and binding-site regions, where available. These features are further described in online supplemental table S1. The scripts used in this study are available at the following GitHub repository: https://github.com/shalawsallah/CACNA1F-variants-analysis.

10.1136/jmedgenet-2020-107404.supp1 Supplementary data

### Machine learning and variant classification

The pathogenicity features that were used to train and validate our prediction model (ProSper) used three different classification algorithms (Hoeffding tree, logit boost and simple logistic) from the machine learning package WEKA (Waikato Environment for Knowledge Analysis^31^) with default parameters. Using a 10-fold cross-validation scheme for the classification of the variants of each gene, the best-performing algorithm was chosen, that is, the algorithm producing the highest MCC value. Prior to this, the supervised instance filters ‘ClassBalancer’ and ‘Discretize’ were applied. The ‘ClassBalancer’ filter compensates for the imbalanced data sets by reweighting the instances in the data and allocating more weight to the variants in the minority class so that each class has the same total weight. For example, in data comprising 20 pathogenic and 80 benign variants, the weight of each of the 20 variants in the minority class is multiplied by 4 in order to create a total weight of 80 in each class. The ‘Discretize’ filter discretises numeric features in the data set into nominal features. For each gene, the exclusion of some features either did not affect model performance or resulted in an increase in performance as measured using MCC. Hence, the minimum number of features was retained while maintaining the highest MCC value for each gene. ‘CorrelationAttributeEval’ from WEKA was used as a guide to identify the more informative features in differentiating between the two classes of variants. ‘CorrelationAttributeEval’ evaluates the worth of a feature by measuring Pearson’s correlation between it and the class. As a result, the number of features included in each model can vary (possible total features=22).

### Gene-specific threshold identification in publicly available tools

Through repeated (n=10) fivefold cross-validation with random subsampling, a subset of the prediction scores (80%) generated in each gene by VEST4, REVEL and ClinPred were used to identify a gene-specific threshold. Using the rest of the prediction scores (20%), an ‘optimised’ MCC value was reported at the newly found gene-specific threshold. The optimised MCC value was then used to evaluate the effectiveness of using the identified threshold in the performance of these three tools. This was later compared with the corresponding original MCC value which was generated using the suggested threshold of 0.5.

## Results

### Identifying a set of X linked genes to evaluate performance of variant interpretation tools

We previously reported a protein-specific or gene-specific approach to variant pathogenicity prediction in the X linked *CACNA1F* gene.^6^ To assess the generalisability of this approach, we again selected X linked disease-causing genes as our test case. From a total of 482 X linked genes from HGMD, no missense variants were reported for 329 genes. Of the remaining 153 genes, we identified 35 which had at least 70 missense pathogenic variants. Of these 35 genes, we found 21 that had a corresponding 3D protein structure or could be modelled using homology modelling. These 21 genes are associated with diseases from multiple organ systems (the genes, reference transcripts and associated disorders are outlined in online supplemental table S2). The gene–disease associations are listed within PanelApp.^32^ The 3D protein structures and homologous templates are shown in online supplemental table S3.

### Data sets

For each gene, data set P comprised missense variants identified from HGMD (5690 variants in total) in addition to nine variants identified as likely pathogenic in our clinical diagnostic laboratory, MGDL. Data set B comprised missense variants identified in gnomAD (1615 variants in total) in addition to four likely benign variants from MGDL. The MGDL variants were classified using the ACMG guidelines. All data set B variants were identified in hemizygous state only and were absent in data set P, that is, the overlaps were removed from data set B. Data sets P and B were considered to represent cohorts that were significantly skewed towards carrying pathogenic and benign variants, respectively. The number of variants in each data set for each gene is shown in online supplemental table S4.

### Protein structures and homology modelling

**Figure 1:**
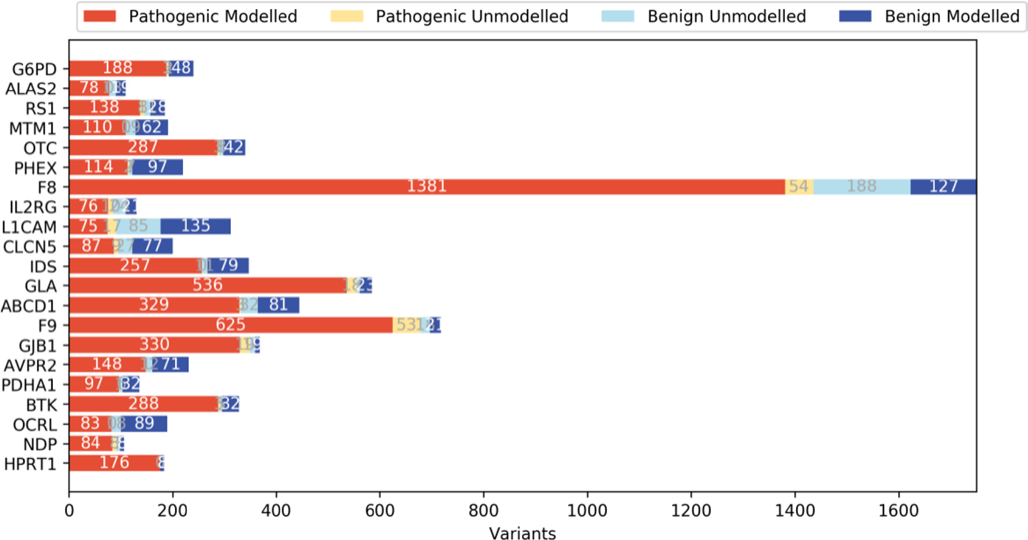
The number of missense variants on the modelled and unmodelled regions from 21 disease-associated X linked genes. Data sets P and B comprised pathogenic and benign variants, respectively. The modelled variants represent variants found on regions with a known structure, or those found in regions shared by both the homologous template and the sequence for the proteins without a structure. The unmodelled variants represent variants outside of these regions.

Experimentally determined structures were available for 8 of 21 proteins analysed. For the rest, we were able to identify appropriate template structures to produce homology models. Templates were chosen if they had 20% sequence identity to the protein of interest and covered at least half of the protein sequence (online supplemental table S3). RaptorX was used in modelling the multi-template *OCRL* (MIM: 300535) and SWISS-MODEL was used to predict the structures of the remaining molecules (online supplemental table S3). The variants identified for each protein were mapped onto the corresponding 3D structure/homologous model and their structural impacts were assessed. Where structural analysis was not possible (ie, for variants on sequences missing from the structure or for those on the sequences that could not be modelled due to the lack of a homologous sequence in the template structure; figure 1 and online supplemental table S5), protein sequence-based analyses such as conservation and amino acid properties were considered.

### Assessing performance of current *in silico* tools to differentiate likely pathogenic from likely benign variants

We assessed the performances of seven previously reported tools in interpreting pathogenicity of missense variants in the 21 studied genes. Assessment was based on ROC and PR AUCs and MCC. In contrast to using MCC, which requires the identification of a pathogenicity threshold for a binary classification, evaluation using the AUCs does not require a single threshold; rather the ROC AUC measures the trade-off between sensitivity and specificity of the classifier over a range of thresholds. The PR AUC measures the trade-off between precision and recall of the classifier. The PR AUC was used to account for the imbalance in numbers between the two classes of variants in data sets P and B. Results for all 21 genes are shown in online supplemental figures S1–S21. It is notable that the ROC and PR AUCs show similar trends for most tools in most (eg, *MTM1* (MIM: 300415) and *OCRL*; online supplemental figures S15 and S16, respectively) but not in all genes (eg, *RS1* (MIM: 300839) and *GLA* (MIM: 300644); online supplemental figures S3 and S8, respectively). In particular, SIFT predictions result in distinctively smaller PR AUCs in 14 genes compared with the other six tools (eg, *GJB1* (MIM: 304040), *F9* (MIM: 300746) and *F8* (MIM: 300841); online supplemental figures S5, S6 and S19, respectively). These differences in the tools’ performances were further studied using MCC values following the assignment of a pathogenicity threshold for each tool (table 1). MCC is a measure of correlation between the observed class of the variants and the predictions made by the tools (a value of 1 represents the highest correlation, −1 represents a negative correlation, and 0 represents no correlation). As part of the analysis based on MCC we found that REVEL, VEST4 and ClinPred were the best-performing tools in interpreting variants in the studied set of 21 genes. Notably, the difference in medians between the prediction scores of these three tools was found to be statistically significant (p<0.002 for each of the 21 genes, Kruskal-Wallis test).

**Table 1:** MCC used to evaluate the performance of the seven tools

Genes	SIFT	PolyPhen2	VEST4	REVEL	ReVe	ClinPred	CAPICE
*G6PD*	0.41	0.42	0.59	**0.61**	0.56	0.52	0.33
*ALAS2*	–	0.41	–	0.63	0.61	**0.73**	0.58
*RS1*	0.38	0.43	**0.69**	0.59	**0.69**	**0.69**	0.43
*MTM1*	0.60	0.67	**0.71**	0.58	0.56	0.66	0.55
*OTC*	0.38	0.38	0.61	0.46	0.46	**0.65**	0.51
*PHEX*	0.42	0.55	**0.62**	0.58	0.48	**0.62**	0.41
*F8*	0.38	0.61	0.75	**0.82**	0.77	0.74	0.63
*IL2RG*	0.52	0.59	0.74	**0.78**	0.61	**0.78**	0.58
*L1CAM*	0.42	0.49	**0.73**	0.65	0.64	0.58	0.51
*CLCN5*	0.55	**0.65**	0.59	0.42	0.56	0.58	0.53
*IDS*	0.62	0.72	0.68	0.64	0.70	**0.79**	0.67
*GLA*	0.26	0.29	**0.57**	0.53	0.51	0.52	0.31
*ABCD1*	0.60	0.63	0.72	0.68	0.69	**0.75**	0.53
*F9*	0.25	0.30	0.39	0.57	**0.59**	0.50	0.41
*GJB1*	0.31	0.35	0.49	**0.67**	0.53	0.53	0.34
*AVPR2*	0.56	0.59	**0.77**	0.62	0.75	0.73	0.65
*PDHA1*	0.62	0.53	0.70	0.60	0.55	**0.80**	0.57
*BTK*	0.69	0.52	**0.80**	**0.80**	0.76	0.72	0.58
*OCRL*	0.54	0.76	**0.78**	0.76	0.68	0.62	0.71
*NDP*	–	0.57	–	0.64	0.59	**0.75**	0.48
*HPRT1*	0.44	0.24	0.73	0.62	0.65	**0.79**	0.48

*As suggested by their respective authors, the pathogenicity thresholds used for SIFT, PolyPhen2, VEST4, REVEL, ReVe, ClinPred and CAPICE were 0.05, 0.85, 0.5, 0.5, 0.7, 0.5 and 0.02, respectively.

†The highest MCC value for each gene is highlighted in bold.

‡The SIFT and VEST4 prediction scores for *ALAS2* and *NDP* variants in the transcript of interest were unavailable.

MCC, Matthews correlation coefficient.

### Assessing variant pathogenicity using a gene-specific approach based on structural analysis

**Figure 2:**
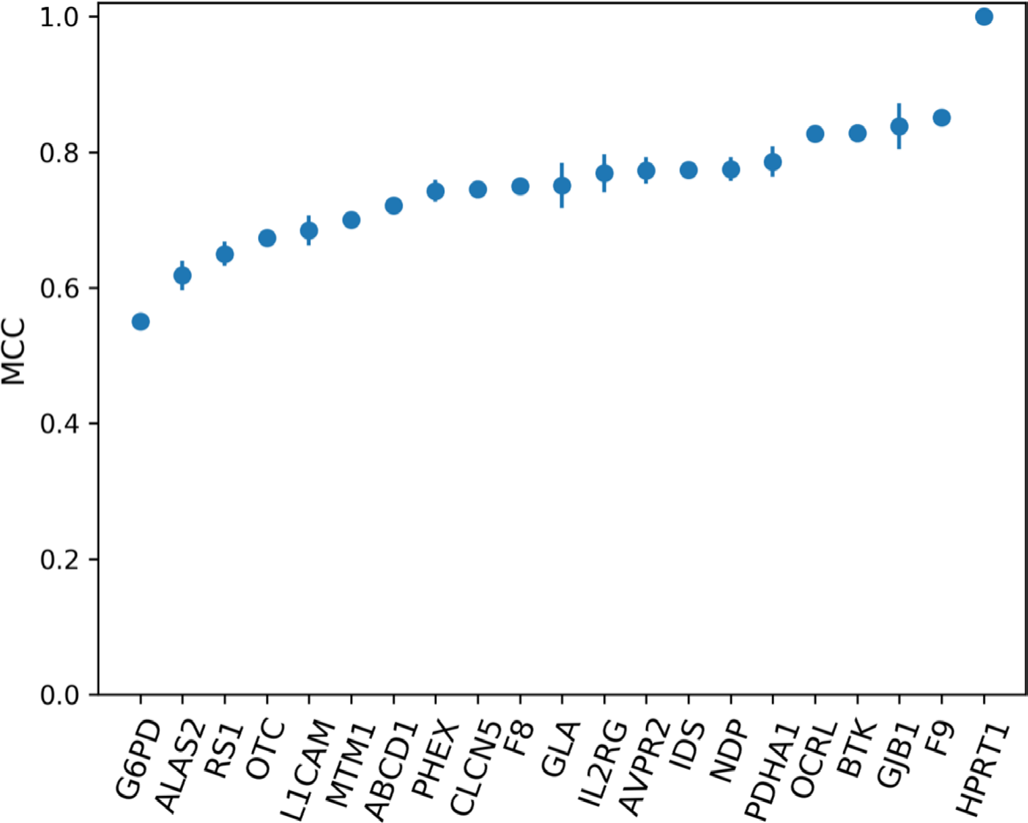
The performance of ProSper (protein-specific variant interpreter) evaluated using Matthews correlation coefficient (MCC) in the classification of variants in 21 genes associated with X linked disorders. For each gene, the line shows the SD from the repeated (n=10) 10-fold cross-validation with random subsampling.

We assessed the accuracy of the gene-specific approach to variant prediction (ProSper) that we developed. ProSper is a binary classifier outputting a prediction probability (figure 2). For each gene, the less informative features were excluded and the corresponding best-performing algorithm was chosen based on MCC values (online supplemental tables S6 and S7). The number of features used to predict pathogenicity ranged from 3 in *G6PD* (MIM: 305900) and *IDS* (MIM: 300823) to 15 features in *CLCN5* (MIM: 300008). The most informative feature was shown to be residue conservation, which was included in 20 of the 21 models. Other informative features included disordered regions, variants’ solvent accessibility, goodness-of-fit test and variants’ impact on protein stability, all of which appeared in at least 14 of the models. The performance of ProSper was evaluated using ROC AUC which ranged from 0.78 to 1, PR AUC which ranged from 0.75 to 1, and MCC which ranged from 0.55 to a perfect correlation of 1 for *G6PD* and *HPRT1* variants, respectively (online supplemental table S8).

### Performance comparison

**Figure 3:**
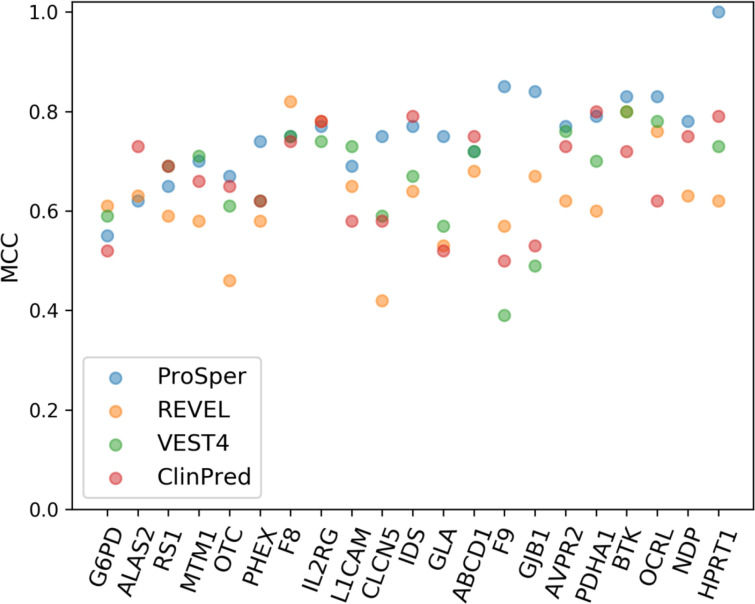
The Matthews correlation coefficient (MCC) values for the gene-specific approach ProSper (protein-specific variant interpreter) compared with REVEL, VEST4 and ClinPred using the complete data sets. The VEST4 MCC results were unavailable as the VEST4 predictions were unavailable for the variants in the transcript of interest in *ALAS2* and *NDP*.

We selected the best-performing tools based on MCC assessment and compared them with ProSper. The gene-specific approach of ProSper produced a higher MCC value in 11 of the 21 genes in comparison with the other three tools (figure 3). ProSper and ClinPred accounted for the highest MCC value for variant prediction in 17 of the 21 genes, with ClinPred outperforming ProSper in predicting variants in 6 of these 21 genes (all 6 with p<0.05 for difference in means, Wilcoxon signed-rank test). In contrast, ProSper outperformed ClinPred in 15 of the 21 genes (p<0.01 for difference in means for 13 genes, Wilcoxon signed-rank test).

### Identifying a gene-specific threshold of pathogenicity for the publicly available tools

**Figure 4:**
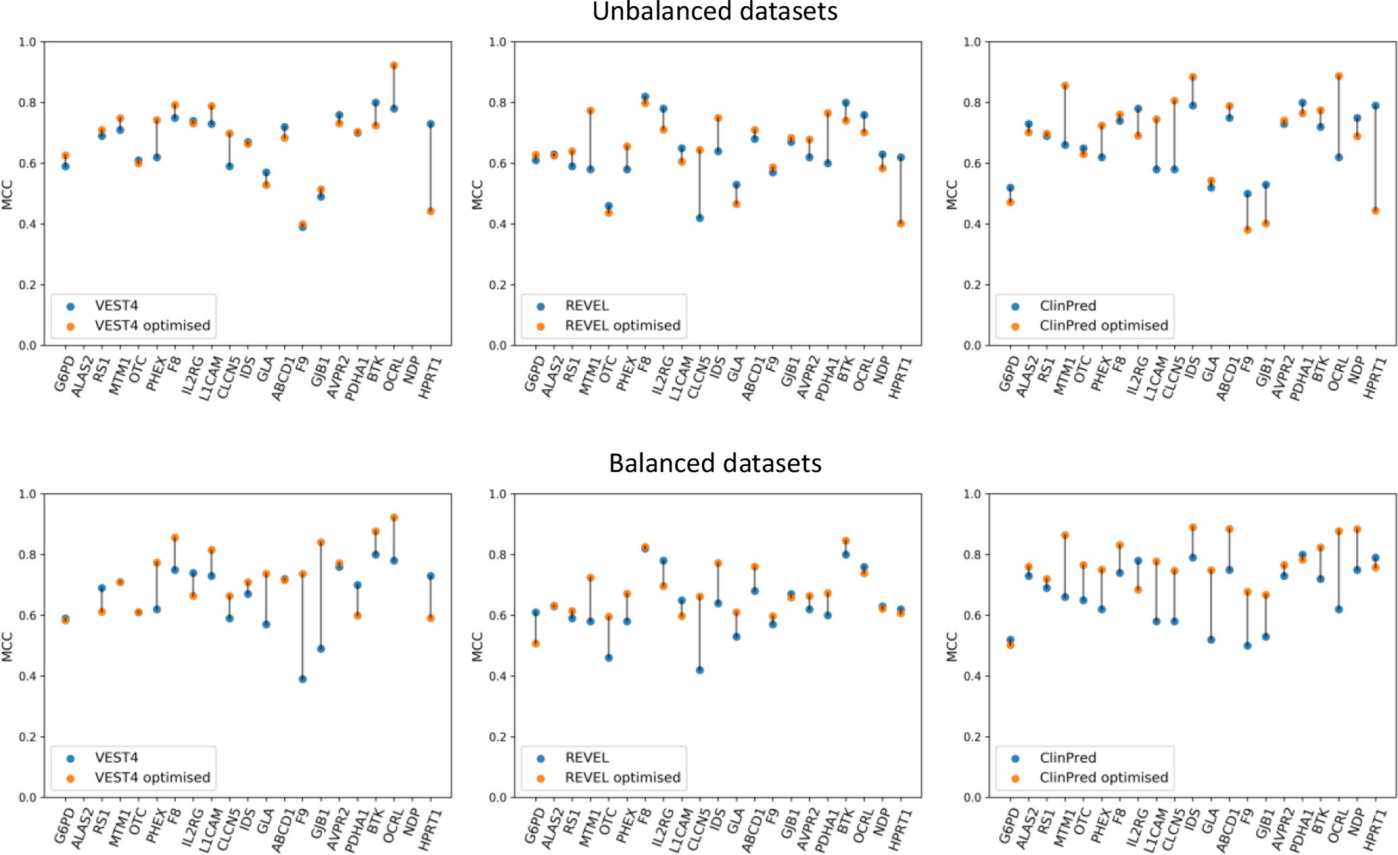
A comparison of the default Matthews correlation coefficient (MCC) with the optimised MCC for the performance of VEST4, REVEL and ClinPred (left, middle and right panels, respectively) using all of the data sets (the top three panels) and using balanced data sets (the bottom three panels) for the 21 genes. For each gene, the data set was balanced using undersampling, that is, using a random subset from the majority class to match the number of variants in the minority class. The default MCC values were generated using the default threshold of 0.5. The optimised MCC values were generated using gene-specific thresholds. The gene-specific threshold was identified using 80% of all the predictions from each tool through repeated (n=10) fivefold cross-validation with random subsampling. The optimised MCC value was generated using the rest (20%) of the predictions from each tool at the threshold identified for each gene. VEST4 predictions were unavailable for *ALAS2* and *NDP* variants in the respective transcripts of interest. The lines between the default MCC and the optimised MCC values for each gene are for visualisation purposes only.

Small changes in the gene-specific classifier threshold can result in large changes to their pathogenicity predictions. Therefore, we tested the hypothesis that the predictions of the best-performing publicly available tools, from the MCC analysis, can be optimised by identifying gene-specific thresholds. From 21 thresholds between 0 and 1, the threshold at which the highest MCC value could be obtained was identified and considered to be the optimum threshold (online supplemental table S9A). The optimum gene-specific thresholds varied widely between genes, ranging from 0.24 to 0.84 for VEST4, from 0.29 to 0.86 for REVEL, and from 0.39 to 0.95 for ClinPred; variability between tools was also observed and the mean value was 0.53, 0.60 and 0.70 for VEST4, REVEL and ClinPred, respectively. Importantly, the resulting optimised MCC values showed an increase in performance for VEST4, REVEL and ClinPred in 11, 11 and 12 different genes, respectively, compared with the original MCC values (figure 4 and online supplemental table S9B).

When evaluating the three tools’ prediction performance using balanced data sets, that is, the same number of variants in both classes as the minority class for each gene (online supplemental table S4), the gene-specific thresholds identified ranged between 0.40 and 0.85, 0.40 and 0.87, and 0.59 and 0.95 for VEST4, REVEL and ClinPred, respectively; the mean value was 0.64, 0.68 and 0.85 for VEST4, REVEL and ClinPred, respectively (online supplemental table S10a). Furthermore, the resulting optimised MCC values showed an increase in performance for VEST4, REVEL and ClinPred in 11, 13 and 17 genes, respectively, compared with the original MCC values (figure 4 and online supplemental table S10b).

**Figure 5:**
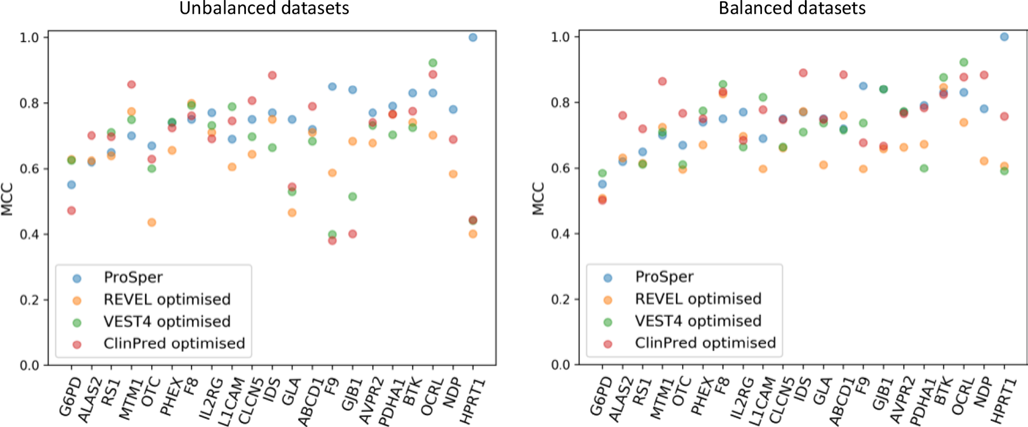
A comparison of the Matthews correlation coefficient (MCC) values for ProSper (protein-specific variant interpreter) with the optimised MCC values for VEST4, REVEL and ClinPred using all of the data sets (on the left) and using balanced data sets (on the right). Optimised MCC values were generated using gene-specific or protein-specific pathogenicity thresholds. The data set for each gene was balanced using undersampling, that is, using a random subset from the majority class to match the number of variants in the minority class. The gene-specific threshold was identified using 80% of all the predictions from each tool through repeated (n=10) fivefold cross-validation with random subsampling. The optimised MCC value was generated using the rest (20%) of the predictions from each tool at the threshold identified for each gene. VEST4 predictions were unavailable for *ALAS2* and *NDP* variants in the respective transcripts of interest.

We compared the optimised performance of the three tools with that of ProSper. Using all of the data available, that is, imbalanced data sets, ProSper outperformed the other three tools in 10 of the 21 genes. Using balanced data sets, the number of genes in which ProSper outperformed the three tools decreased to four (figure 5). ProSper outperformed the optimised ClinPred in 7 genes using balanced data sets, compared with 12 genes using imbalanced data sets. The optimised ClinPred was superior to ProSper in 11 genes using balanced data sets, compared with 9 genes using imbalanced data sets.

## Discussion

This study focuses on the analysis of missense changes in genes associated with X linked recessive disorders. We used internally generated and publicly available variant data sets to evaluate existing bioinformatic tools that assess variant pathogenicity. We found that using a gene-specific threshold for these algorithms results in a better performance in at least 50% of the studied genes. We also developed and trained ProSper, a protein-specific variant interpreter that combines genetic and protein structural data. This tool performed strongly and was found to be better than established methods in around 50% of the studied genes.

Of seven publicly available missense prediction tools, ClinPred, VEST4 and REVEL almost always outperformed other tools for the 21 genes investigated (table 1). This is in keeping with previous reports that have found REVEL and VEST3 (the previous version of VEST4) to be among the most accurate missense prediction tools available.^18 19^ However, the findings of other reports showing ReVe^19^ and CAPICE^20^ to be performing strongly could not be replicated for this subset of genes using this data set. Notably, ProSper outperformed all seven tools in 11 of the 21 genes assessed.

Identifying gene-specific thresholds rather than universal thresholds resulted in a better performance for VEST4, REVEL and ClinPred (figure 4 and online supplemental table S9b). The lack of improvement in performance in some genes is probably due to the default threshold being optimal. The drop in performance in other genes, including *NDP* (MIM: 300658) and *HPRT1* (MIM: 308000), when a gene-specific threshold was used was likely due to the effect of fivefold cross-validation on a small and/or an imbalanced data set; this can lead to a threshold that is highly skewed by the majority class. Overall, our data support that using a gene-specific pathogenicity thresholds can optimise these tools’ performance. Such an approach would result in these three tools outperforming ProSper in more genes (figure 5). These observations support others’ findings.^33–36^ It has been suggested that the use of gene-specific thresholds may be necessary if the strength of evidence provided by computational tools is to be increased from ‘supporting’ to ‘moderate’ in the ACMG guidelines.^18^

ProSper’s performance is not constrained by the input information, suggesting that ProSper can be widely applied. First, ProSper’s performance was similar when using experimentally determined structures compared with when using homology models despite the latter being less accurate. Intriguingly, ProSper’s lowest performance was for four genes where experimentally determined protein structures were used. It is worth noting that ProSper performed equally well where homologous models with relatively low sequence identity between the protein of interest and the template were used and in models where the proteins’ sequence coverage was relatively low. Second, ProSper’s performance appeared to be independent of the number of variants for each gene and of the ratio of pathogenic to benign variants, for example, *ALAS2* (MIM: 301300), *NDP* and *HPRT1*. Finally, ProSper used as few as three features to predict variants to achieve a very good performance in a subset of genes (including an MCC value of 0.55 and 0.77 for *G6PD* and *IDS*, respectively). Overall, these observations underline the value of gene-specific analysis in variant interpretation.^37 38^

Gene-specific approaches may be limited by the number of variants available for each gene. ProSper is likely to perform better in the presence of larger and more balanced data sets, although our data from *HPRT1* and *NDP* suggest this might not be always necessary. Also, ProSper requires a protein structure or a homologous model that covers variants of interest. This is lacking for some molecules, although the availability of structural data is increasing over time; for example, of 66 X linked genes in HGMD^10^ V.2020.4 with 30 or more missense variants we found 50 which had a structure or could be modelled (>20% sequence identity and >40% protein sequence coverage; data not shown), demonstrating the potential applicability of this approach as the number of reported variants increases. Notably, it was difficult to structurally analyse ‘unmodelled’ variants, that is, those missing from the protein structure (indicating disordered regions of the proteins which are difficult to determine) and variants found outside of the modelled regions (indicating regions lacking sequence conservation). However, in 11 of the 21 genes, whether or not a variant could be modelled was a strong indicator of variant pathogenicity with most unmodelled variants being benign. Moreover, ProSper’s performance was relatively accurate in genes with structures/models covering only 58%–64% of the protein sequence (MCC=0.69, MCC=0.77 and MCC=0.75 for variant classification in *L1CAM* (MIM: 308840), *IL2RG* (MIM: 308380) and *F8*, respectively) and in genes with the lowest proportion of modelled variants (MCC=0.69, MCC=0.77 and MCC=0.75 for variant classification in *L1CAM, IL2RG* and *CLCN5*, respectively). It is worth noting that this study is limited by the difficulty in definitively assigning variants to either pathogenic or benign classes. There is a broad effort from researchers and clinicians to apply most up-to-date guidelines when reporting, annotating and depositing variants in ClinVar database,^39^ which in combination with HGMD and gnomAD is becoming a robust resource to exclude variants with conflicting evidence/annotations. Finally, it was difficult to account for information leakage, that is, the evaluation of the publicly available tools using variants in our data set which were used to train these tools initially.^40^ Information leakage likely inflates the true performance of some of these prediction tools. Filtering the data set to only include recently reported variants can minimise this problem. However, this results in the exclusion of a large proportion of the data set which constrains a gene-specific classification if the same data set is to be used in comparing all tools.

In conclusion, the gene-specific approach that we developed (ProSper) often outperformed currently available tools in evaluating the pathogenicity of missense variants; this was the case for 11 of the 21 X linked disease-implicated genes that met the inclusion criteria for this study. In addition, analysis of previously reported algorithms revealed that REVEL, VEST4 and ClinPred were relatively consistent and accurate for this group of molecules. Generally, using gene-specific pathogenicity thresholds increased the prediction performance for all these three tools. Despite the gene-dependent optimisation of VEST4, REVEL and ClinPred, ProSper outperformed all tools for some genes. Hence, the gene-specific structural analysis underlying ProSper can contribute to the improvement of variant interpretation, enabling precise and timely diagnosis.

## Data Availability

Some of the data that support the findings of this study are available from gnomAD, a public open access repository, at http://gnomad.broadinstitute.org. Some of the data are available from HGMD at https://portal.biobase-international.com/cgi-bin/portal/login.cgi and restrictions apply to the availability of these data, which are used under licence for this study. The rest of the data from the Manchester Genomic Diagnostic Laboratory are not publicly available due to privacy or ethical restrictions, but are available on request from the corresponding author.
