# Supplementary material for: Improving the clinical interpretation of missense variants in X linked genes using structural analysis
Source: J Med Genet. 2021 Mar 25;59(4):385–92. doi: 10.1136/jmedgenet-2020-107404 (PMC8961765; doi:10.1136/jmedgenet-2020-107404)
Supplement: Supplementary data [file jmedgenet-2020-107404supp001.pdf]

Table S1. A list of the features retrieved and used to annotate the variants for each of the 21 genes. The general features were retrieved in all genes, whereas the gene-specific features were only available in certain genes.

**Variant features considered in all genes (general features)**
i) Protein variants within sequences with a known 3D structure. Across the 21 genes, the majority of pathogenic variants lie within modelled regions (i.e. regions with a known structure or regions shared between the homologous template and the protein sequence) compared to the benign variants.<br>*ii) Residue-volume and molecular goodness-of-fit.* All low-energy side chain conformations were assessed and their "goodness-of-fit" evaluated to determine the steric clashes with the neighbouring residues created through replacing smaller residues with larger ones. It was hypothesized that a larger number of dataset P variants create more steric clashes indicating the introduction of van der Waals' overlaps between the close atoms. This was expected to correlate with the results found in volume-change where a higher number of dataset P variants result in the replacement of smaller residues with larger ones. Replacing larger residues with smaller ones, especially in the core of a protein is also expected to destabilise protein structure. Therefore, the variants leading to the disruption of packing interactions are more likely to be pathogenic.<br>*iii) Variants altering charged residues.* Alterations in both negatively and positively charged residues in certain proteins like ion channels are likely to affect function. The loss of charged residues on the surface of a protein may lead to disruption of intermolecular as well as intramolecular interactions. Similarly, the introduction of charged residues to the core of a protein is likely to disrupt hydrophobic interactions and destabilise the protein.<br>*iv) Variants altering hydrophobic residues.* Replacing a hydrophobic residue with a hydrophilic one, especially in a transmembrane region or core of a protein, can result in loss of hydrophobic

interactions leading to structural instability. Similarly, introduction of a hydrophobic residue on the surface of a protein can make protein aggregation more likely.

v) Side-chain solvent accessibility. Residues can be hidden in the core of the protein or be solvent accessible on the surface. Measuring solvent accessibility to each residue allows for the categorization of the residues from completely hidden to fully accessible on the surface and anywhere in between. The dataset P variants in most of the proteins were identified to be towards the core of the protein rather than on the surface.

vi) Conservation at variant's site. The loss of a conserved residue is more likely to be detrimental to the structure and function of the protein. In particular, when the change is less conservative, i.e. replacement of a residue with another with very different physicochemical properties. The dataset P variants are expected to result in the loss of more conserved residues compared to the dataset N variants.

vii) Alteration in residues with special physicochemical properties. Variants involving glycine, proline, and cysteine were considered to more likely affect protein structure. Glycine is the smallest of the residues and replacing it in the core of a protein with any of the larger residues can create stress on the structure and destabilize the protein. The introduction of glycine into the core of a protein to replace one of the larger residues can also result in instability. Similarly, replacing proline with most other residues, or vice versa, is more likely to be destabilizing due to its unique ring-shaped structure. Proline can introduce a turn in the structure, such as in tight turns, which other residues can't replicate. The loss or gain of cysteine was also considered where surface/extracellular variants are likely to lead to the loss or formation of disulphide bonds, respectively, causing protein instability.

viii) Disease propensity at the site of variant. The loss of residues previously associated with the disease are more likely to be pathogenic when mutated. This is equivalent to the residue having mutated more than once in the dataset for each protein.

ix) The secondary structure of the site of variant. Beta strands or alpha helices can be less tolerant to certain changes compared to loops. The introduction of a proline onto a beta strand, for instance, is likely to effectively break the strand and disturb the hydrogen bonds forming a beta sheet, whereas the same change may be more tolerated on an alpha helix.<br>*x) Effect on protein-protein interaction and protein stability*<br>The likelihood of the site of the variant being involved in protein-protein interaction were predicted using an external tool. The effect of the variants on protein stability was also predicted. More of the disease-implicated variants are likely to destabilize the protein structure or be involved in interactions.<br>*xi) Disorders regions.* Disordered regions are more flexible in nature and promiscuous in their ability to bind proteins. Variants in these regions can easily disturb this finely tuned region resulting in more sensitive and less specific, i.e. non-functional, binding which can lead to binding disruption or aggregation.
**Variant features considered in certain genes (gene-specific features)**
i) Variant clustering. In the proteins where variant-clustering was noted in dataset P in comparison to dataset B through visual inspection. The protein was either divided into halves by drawing a plain through the centre of its mass or it was separated into multiple protein/functional domains. Variant clustering based on the secondary structure was also considered.<br>*ii) Functional site.* Variations at functional sites, including binding sites, are more likely to affect protein function.

Table S2. A list of the 21 X-linked genes included in this study and the diseases associated with these genes. The transcripts were those chosen by the Human Gene Mutation Database.

Genes	Transcript	Associated Disorders	Phenotype MIM
G6PD	ENST00000393564	Haemolytic anaemia	300908
ALAS2	ENST00000650242	Sideroblastic anaemia 1, Erythropoietic protoporphyria	300751, 300752
RS1	ENST00000379984	Retinoschisin	312700
MTM1	ENST00000370396	Myotubular myopathy	310400
OTC	ENST00000039007	Ornithine transcarbamylase deficiency	311250
PHEX	ENST00000379374	Hypophosphatemic rickets	307800
F8	ENST00000360256	Hemophilia A	306700
IL2RG	ENST00000374202	Moderate combined immunodeficiency, Severe combined immunodeficiency	312863, 300400
L1CAM	ENST00000370060	Partial agenesis of corpus callosum, CRASH syndrome, Hydrocephalus, MASA syndrome	304100, 303350, 307000, 303350
CLCN5	ENST00000307367	Dent disease, Hypophosphatemic rickets, Nephrolithiasis I, Proteinuria	300009, 300554, 310468, 308990
IDS	ENST00000340855	Mucopolysaccharidosis II	309900
GLA	ENST00000218516	Fabry disease (systemic)	301500
ABCD1	ENST00000218104	Adrenomyeloneuropathy	300100
F9	ENST00000218099	Hemophilia B	306900
GJB1	ENST00000361726	Charcot-Marie-Tooth neuropathy	302800
AVPR2	ENST00000337474	Nephrogenic diabetes insipidus, Nephrogenic syndrome inappropriate antidiuresis	304800, 300539
PDHA1	ENST00000422285	Pyruvate dehydrogenase E1-α deficiency	312170
BTK	ENST00000308731	Agammaglobulinemia, Isolated GH deficiency III with agammaglobulinemia	300755, 307200
OCRL	ENST00000371113	Lowe syndrome, Dent disease 2	309000, 300555
NDP	ENST00000642620	Norrie disease, Exudative vitreoretinopathy 2	310600, 305390
HPRT1	ENST00000298556	Lesch-Nyhan syndrome, Gout (HPRT)	300322, 300323

Table S3. The experimentally-solved structures and the homologous templates used in analyzing the missense variants of the 21 genes. An available protein structure (s) was used for variant analysis in some the proteins, whereas a homologous model (m) was used for those proteins without a solved structure.

Genes	Structure(s)/Model(m)	Sequence identity (%)	Sequence coverage (%)
G6PD	2BHL(s)	-	95
ALAS2	5QQQ(s)	-	77
RS1	3JD6(s)	-	75
MTM1	1M7R(m)	67	95
OTC	1FVO(s)	-	90
PHEX	4CTH(m)	37	91
F8	6MF2(m)	97	64
IL2RG	2ERJ(m)	99	63
L1CAM	3DMK(m)	22	58
CLCN5	6QVB(m)	28	83
IDS	6IOZ(s)	-	93
GLA	1R47(s)	-	92
ABCD1	6JBJ(m)	27	78
F9	5EDM(m)	34	89
GJB1	2ZW3(m)	66	76
AVPR2	4ZJH(m)	24	83
PDHA1	3EXH(m)	100	93
BTK	4Y93(m), 4XI2(m)	97	94
OCRL	2QV2(m), 3QBT(m), 2KIE(m), 4CMN(m)	100	90
NDP	5BQ8(s)	-	82
HPRT1	1BZY(s)	-	100

Table S4. The missense variants identified from 21 disease-associated X-linked genes. Dataset P and B comprised pathogenic and benign variants, respectively. The variants which appeared in both datasets, i.e. overlapping variants, were removed from dataset B.

Genes	Number of variants in dataset P	Number of variants in dataset B	Overlapping dataset B variants removed	Total
G6PD	191	50	43	241
ALAS2	78	32	7	110
RS1	146	39	2	185
MTM1	110	81	0	191
OTC	290	50	7	340
PHEX	116	104	4	220
F8	1435	315	71	1750
IL2RG	86	45	0	131
L1CAM	92	220	7	312
CLCN5	96	104	3	200
IDS	257	90	8	347
GLA	554	30	26	584
ABCD1	332	113	7	445
F9	678	39	16	717
GJB1	349	19	15	368
AVPR2	149	83	7	232
PDHA1	98	38	3	136
BTK	291	37	2	328
OCRL	83	107	1	190
NDP	92	15	3	107
HPRT1	176	8	3	184

Table S5. The pathogenic and benign variants on the modelled and the unmodelled regions. The modelled variants represent variants found on regions with a known structure, or those found in regions shared by both the homologous template and the protein sequence for those proteins without a solved structure. The unmodelled variants represent variants outside of these regions.

Genes	Pathogenic variants modelled	Benign variants modelled	Pathogenic variants unmodelled	Benign variants unmodelled	All variants modelled (%)
G6PD	188	48	3	2	98
ALAS2	78	19	0	13	88
RS1	138	28	8	11	90
MTM1	110	62	0	19	90
OTC	287	42	3	8	97
PHEX	114	97	2	7	96
F8	1381	127	54	188	86
IL2RG	76	21	10	24	74
L1CAM	75	135	17	85	67
CLCN5	87	77	9	27	82
IDS	257	79	0	11	97
GLA	536	23	18	7	96
ABCD1	329	81	3	32	92
F9	625	21	53	18	90
GJB1	330	9	19	10	92
AVPR2	148	71	1	12	94
PDHA1	97	32	1	6	95
BTK	288	32	3	5	98
OCRL	83	89	0	18	91
NDP	84	8	8	7	86
HPRT1	176	8	0	0	100

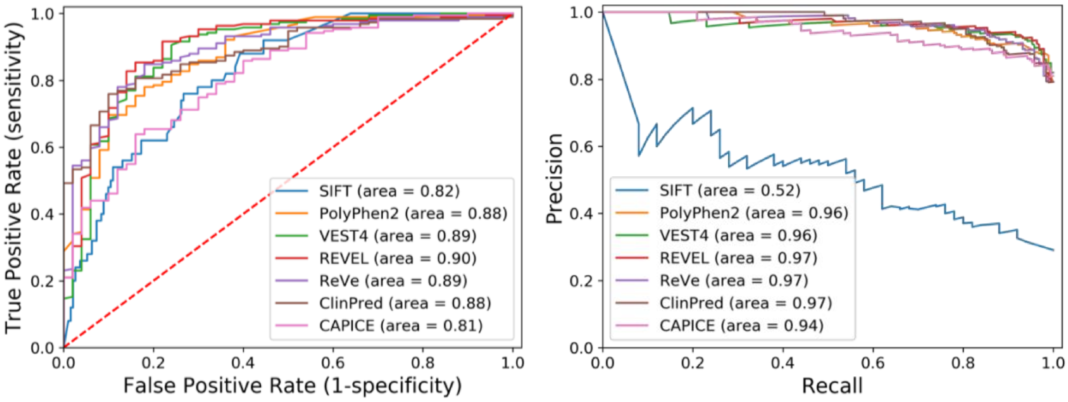

Figure S1. The area under the receiver operating characteristic (ROC) curve (left panel) and the precision recall (PR) curve (right panel) of different prediction tools in classifying *G6PD* variants (191 pathogenic and 50 benign variants).

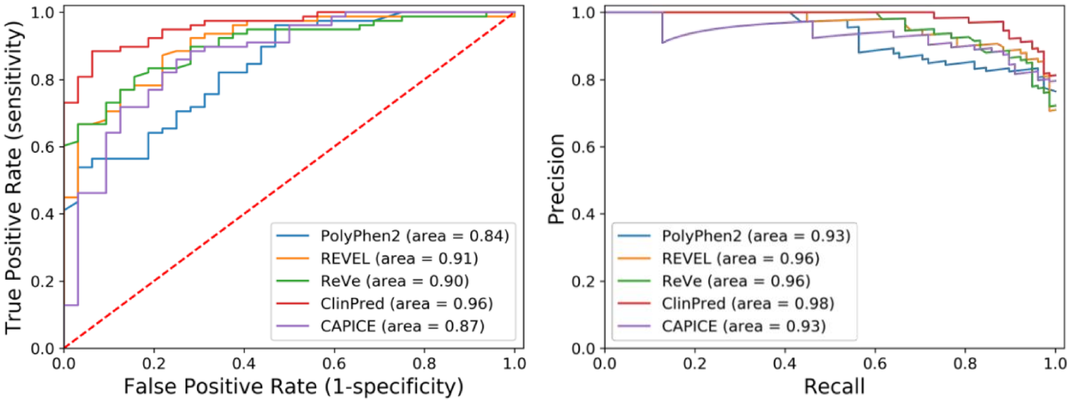

Figure S2. The area under the receiver operating characteristic (ROC) curve (left panel) and the precision recall (PR) curve (right panel) of different prediction tools in classifying *ALAS2* variants. SIFT and VEST4 predictions were unavailable for the variants in the transcript of interest (78 pathogenic and 32 benign variants).

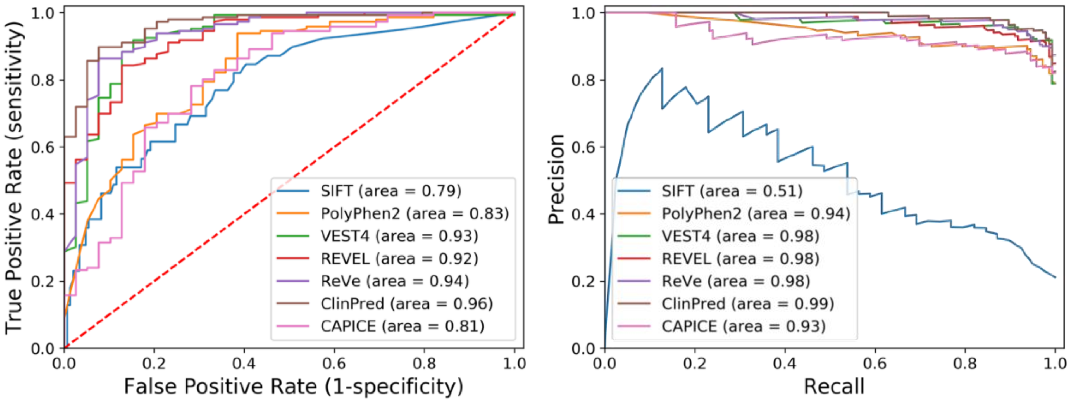

Figure S3. The area under the receiver operating characteristic (ROC) curve (left panel) and the precision recall (PR) curve (right panel) of different prediction tools in classifying *RS1* variants (146 pathogenic and 39 benign variants).

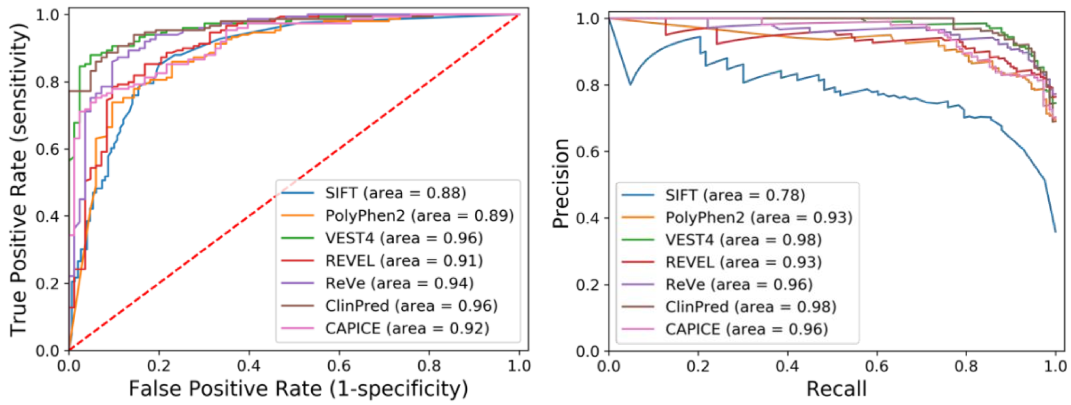

Figure S4. The area under the receiver operating characteristic (ROC) curve (left panel) and the precision recall (PR) curve (right panel) of different prediction tools in classifying *AVPR2* variants (149 pathogenic and 83 benign variants).

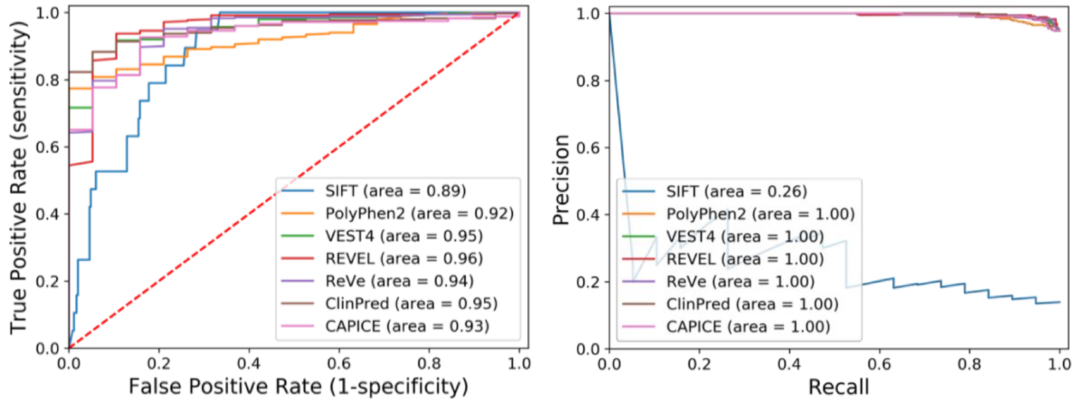

Figure S5. The area under the receiver operating characteristic (ROC) curve (left panel) and the precision recall (PR) curve (right panel) of different prediction tools in classifying *GJB1* variants (349 pathogenic and 19 benign variants).

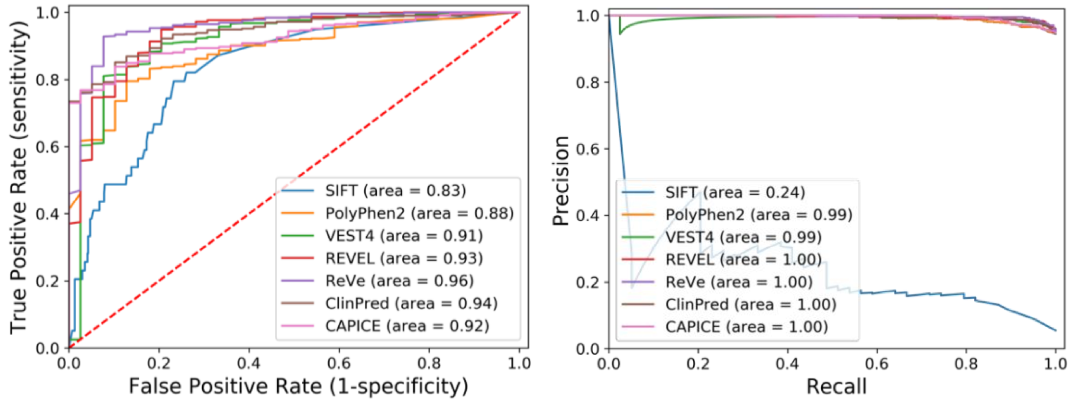

Figure S6. The area under the receiver operating characteristic (ROC) curve (left panel) and the precision recall (PR) curve (right panel) of different prediction tools in classifying *F9* variants (678 pathogenic and 39 benign variants).

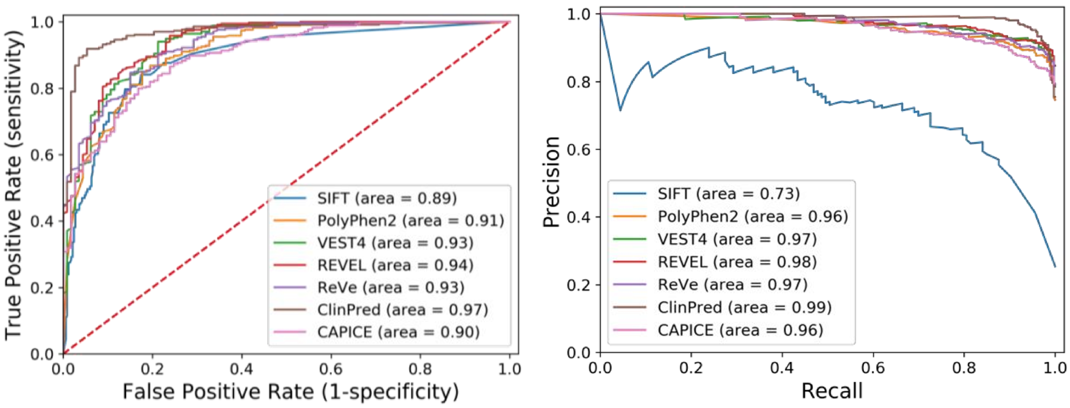

Figure S7. The area under the receiver operating characteristic (ROC) curve (left panel) and the precision recall (PR) curve (right panel) of different prediction tools in classifying *ABCD1* variants (332 pathogenic and 113 benign variants).

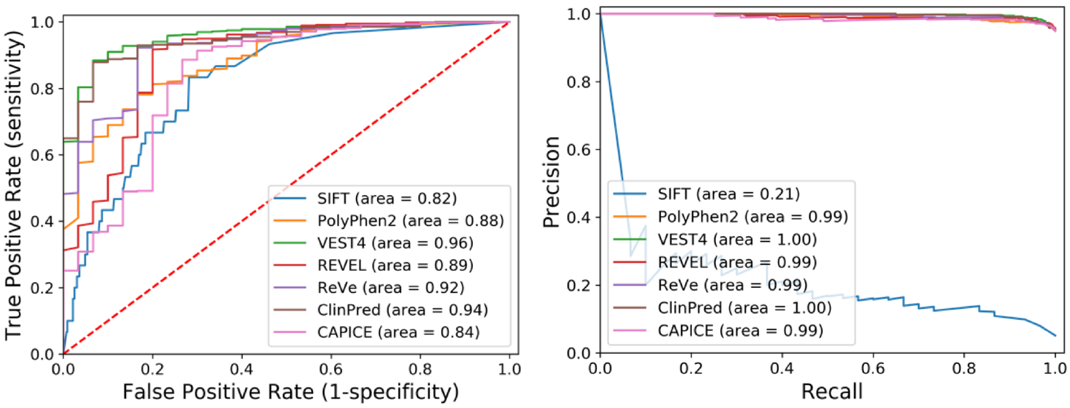

Figure S8. The area under the receiver operating characteristic (ROC) curve (left panel) and the precision recall (PR) curve (right panel) of different prediction tools in classifying *GLA* variants (554 pathogenic and 30 benign variants).

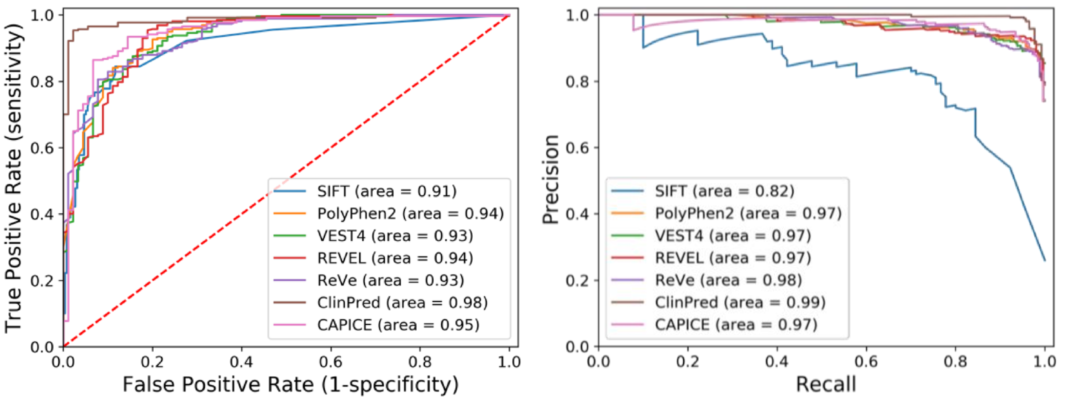

Figure S9. The area under the receiver operating characteristic (ROC) curve (left panel) and the precision recall (PR) curve (right panel) of different prediction tools in classifying *IDS* variants (257 pathogenic and 90 benign variants).

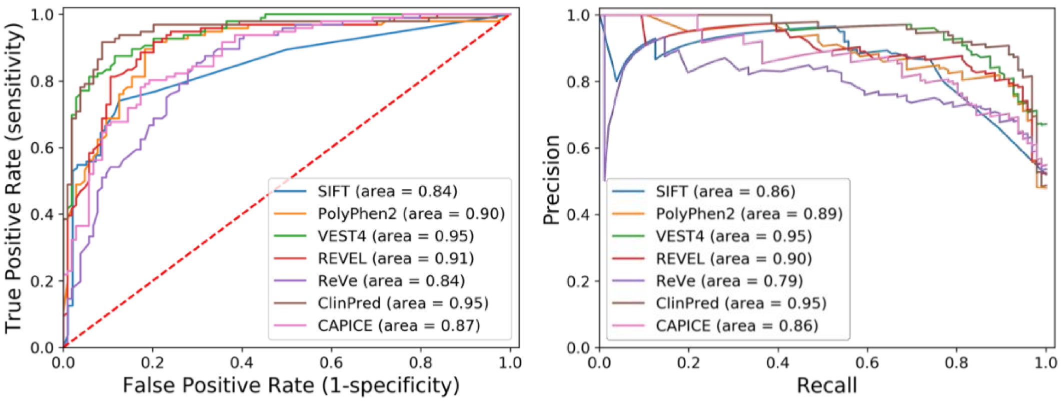

Figure S10. The area under the receiver operating characteristic (ROC) curve (left panel) and the precision recall (PR) curve (right panel) of different prediction tools in classifying *CLCN5* variants (96 pathogenic and 104 benign variants).

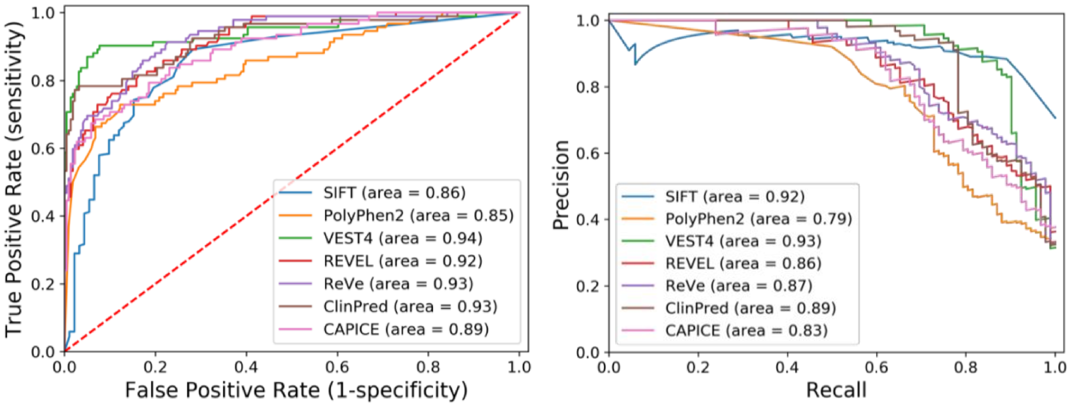

Figure S11. The area under the receiver operating characteristic (ROC) curve (left panel) and the precision recall (PR) curve (right panel) of different prediction tools in classifying *L1CAM* variants (92 pathogenic and 220 benign variants).

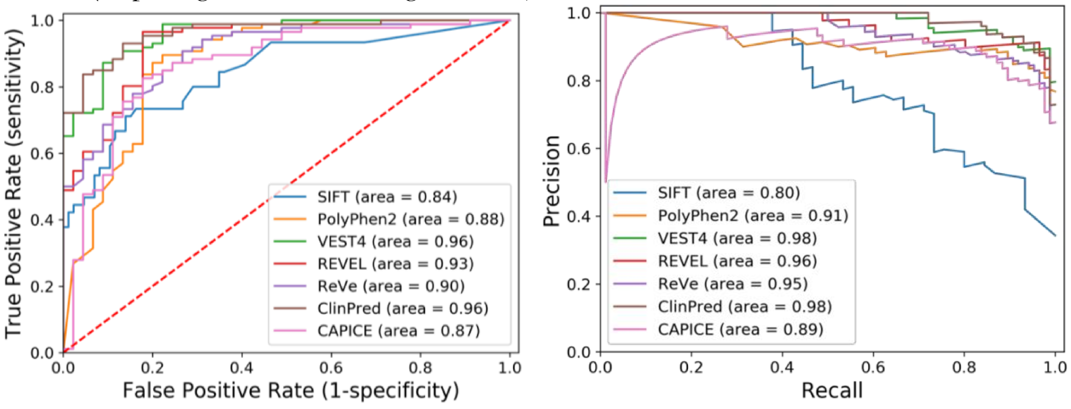

Figure S12. The area under the receiver operating characteristic (ROC) curve (left panel) and the precision recall (PR) curve (right panel) of different prediction tools in classifying *IL2RG* variants (86 pathogenic and 45 benign variants).

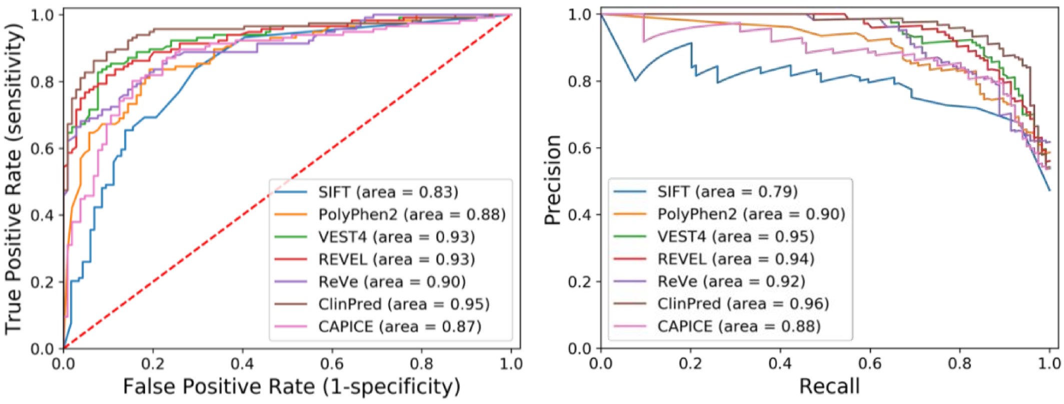

Figure S13. The area under the receiver operating characteristic (ROC) curve (left panel) and the precision recall (PR) curve (right panel) of different prediction tools in classifying *PHEX* variants (116 pathogenic and 104 benign variants).

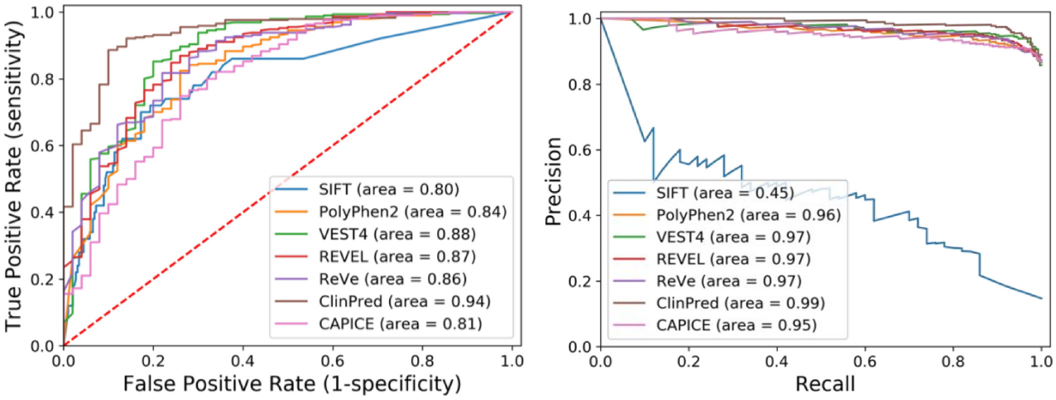

Figure S14. The area under the receiver operating characteristic (ROC) curve (left panel) and the precision recall (PR) curve (right panel) of different prediction tools in classifying *OTC* variants (290 pathogenic and 50 benign variants).

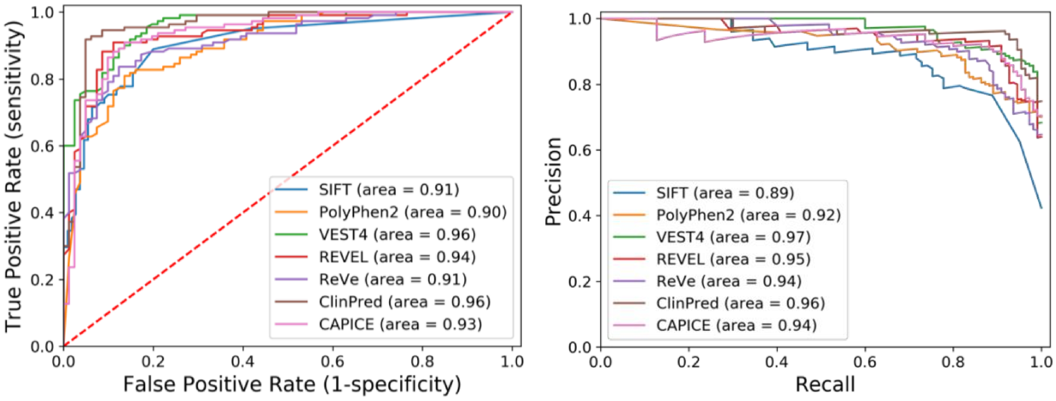

Figure S15. The area under the receiver operating characteristic (ROC) curve (left panel) and the precision recall (PR) curve (right panel) of different prediction tools in classifying *MTM1* variants (110 pathogenic and 81 benign variants).

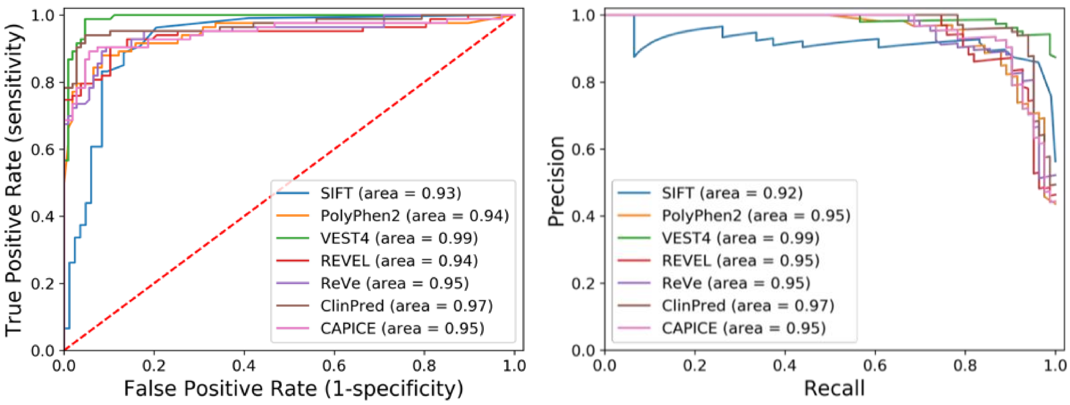

Figure S16. The area under the receiver operating characteristic (ROC) curve (left panel) and the precision recall (PR) curve (right panel) of different prediction tools in classifying *OCRL* variants (83 pathogenic and 107 benign variants).

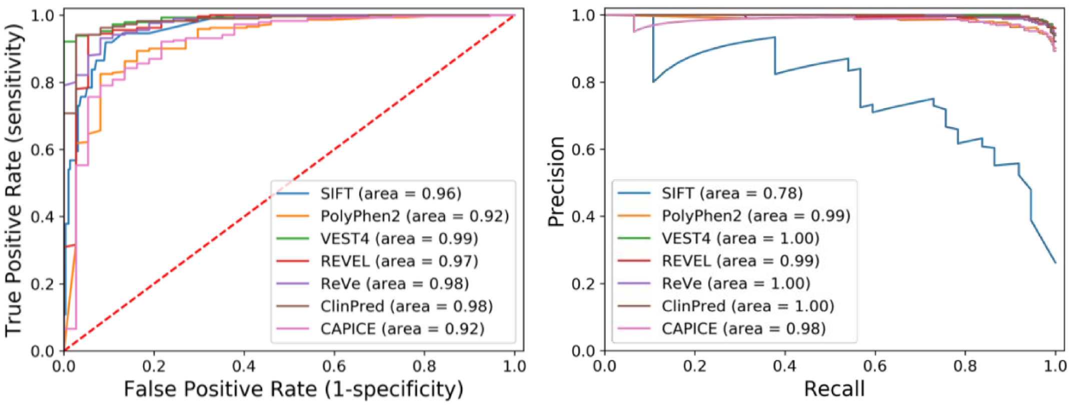

Figure S17. The area under the receiver operating characteristic (ROC) curve (left panel) and the precision recall (PR) curve (right panel) of different prediction tools in classifying *BTK* variants (291 pathogenic and 37 benign variants).

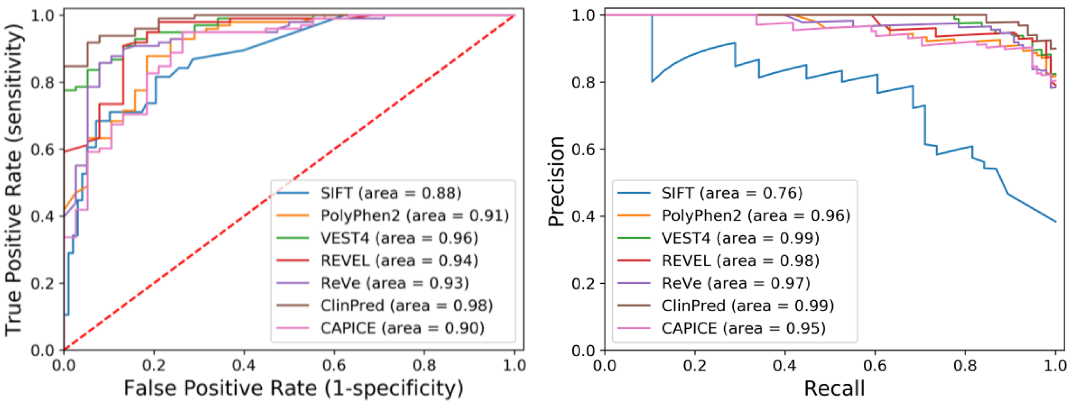

Figure S18. The area under the receiver operating characteristic (ROC) curve (left panel) and the precision recall (PR) curve (right panel) of different prediction tools in classifying *PDHA1* variants (98 pathogenic and 38 benign variants).

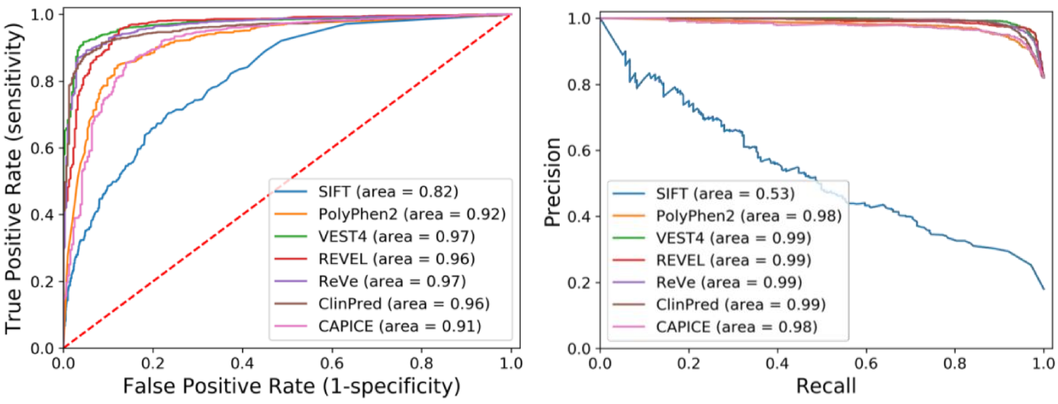

Figure S19. The area under the receiver operating characteristic (ROC) curve (left panel) and the precision recall (PR) curve (right panel) of different prediction tools in classifying *F8* variants (1435 pathogenic and 315 benign variants).

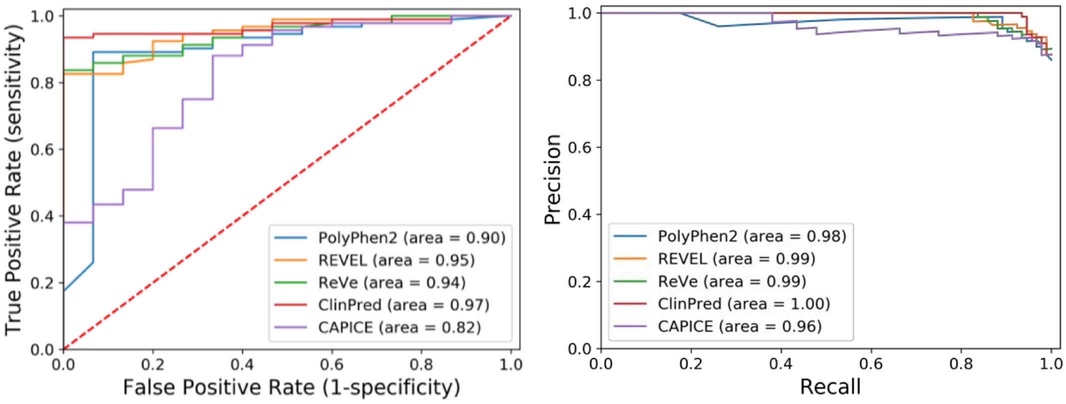

Figure 20. The area under the receiver operating characteristic (ROC) curve (left panel) and the precision recall (PR) curve (right panel) of different prediction tools in classifying *NDP* variants (92 pathogenic and 15 benign variants). SIFT and VEST4 predictions were unavailable for the variants in the transcript of interest.

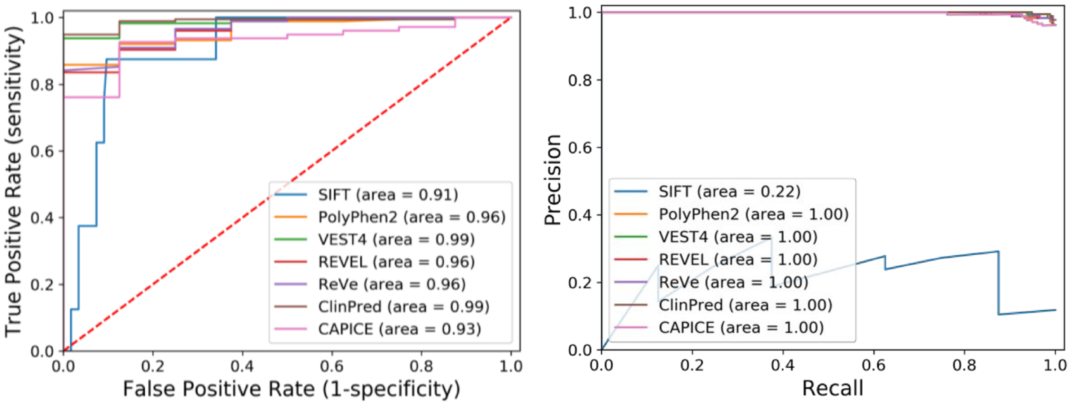

Figure S21. The area under the receiver operating characteristic (ROC) curve (left panel) and the precision recall (PR) curve (right panel) of different prediction tools in classifying *HPRT1* variants (176 pathogenic and 8 benign variants).

Table S6. The three algorithms used in building ProSper. A minimum number of features were retained, *i.e.* selected features, while maintaining the highest possible performance as measured by the Matthews Correlation Coefficient (MCC) test. The dropped features either did not impact model performance or increased model performance.

Genes	WEKA algorithm	Number of features considered	Number of features selected
G6PD	Logit Boost	17	3
ALAS2	Hoeffding Tree	17	5
RS1	Logit Boost	18	9
MTM1	Hoeffding Tree	18	6
OTC	Hoeffding Tree	19	9
PHEX	Logit Boost	19	5
F8	Logit Boost	20	8
IL2RG	Hoeffding Tree	18	13
L1CAM	Simple Logistic	19	4
CLCN5	Hoeffding Tree	19	15
IDS	Hoeffding Tree	18	3
GLA	Simple Logistic	18	9
ABCD1	Logit Boost	20	6
F9	Hoeffding Tree	19	6
GJB1	Hoeffding Tree	18	4
AVPR2	Logit Boost	18	9
PDHA1	Hoeffding Tree	17	12
BTK	Hoeffding Tree	20	5
OCRL	Hoeffding Tree	21	4
NDP	Hoeffding Tree	17	10
HPRT1	Hoeffding Tree	19	6

Table S7. The most informative features identified for each gene in developing ProSper.

Genes	Features selected
G6PD	Conservation, solvent accessibility, predicted to impact protein stability
ALAS2	Reference amino acid, residue number, on modelled regions, conservation, predicted to impact protein stability
RS1	On modelled regions, conservation, loss or gain of charged residues, loss or gain of proline, loss or gain of cysteine, solvent accessibility, goodness-of-fit, predicted to impact protein stability, predicted to impact protein-protein interaction
OTC	On modelled regions, conservation, loss or gain of proline, disordered region, solvent accessibility, goodness-of-fit, loss or gain of charged residues, predicted to impact protein stability, predicted to impact protein-protein interaction
L1CAM	Reference amino acid, alternative amino acid, conservation, loss or gain of hydrophobic residues
MTM1	Residue number, conservation, disordered region, solvent accessibility, goodness-of-fit, predicted to impact protein stability
ABCD1	Residue number, on modelled regions, conservation, loss or gain of proline, disordered region, solvent accessibility
PHEX	Alternative amino acid, conservation, goodness-of-fit, solvent accessibility, predicted to impact protein stability
CLCN5	Reference amino acid, alternative amino acid, residue number, topology, on modelled regions, conservation, secondary structure, disordered region, solvent accessibility, goodness-of-fit, change in residue volume, loss or gain of charged residues, predicted to impact protein stability, predicted to impact protein-protein interaction, loss or gain of glycine
F8	On modelled regions, conservation, disordered region, solvent accessibility, goodness-of-fit, loss or gain of hydrophobic residues, change in residue volume, predicted to impact protein stability
GLA	On modelled regions, conservation, disordered region, solvent accessibility, goodness-of-fit, loss or gain of hydrophobic residues, change in residue volume, predicted to impact protein stability, predicted to impact protein-protein interaction

IL2RG	Alternative amino acid, topology, on modelled regions, conservation, loss or gain of cysteine, disordered region, solvent accessibility, goodness-of-fit, change in residue volume, loss or gain of charged residues, predicted to impact protein stability, predicted to impact protein-protein interaction, loss or gain of hydrophobic residues
AVPR2	Reference amino acid, residue number, conservation, topology, disordered region, goodness-of-fit, solvent accessibility, loss or gain of charged residues, predicted to impact protein stability
IDS	Residue number, conservation, goodness-of-fit
NDP	Reference amino acid, alternative amino acid, residue number, on modelled regions, conservation, disordered region, goodness-of-fit, glycine, loss or gain of cysteine, change in residue volume
PDHA1	Reference amino acid, alternative amino acid, residue number, on modelled regions, conservation, secondary structure, disordered region, change in residue volume, goodness-of-fit, solvent accessibility, predicted to impact protein stability, predicted to impact protein-protein interaction
OCRL	Disordered region, variant clustering region, conservation, predicted to impact protein stability
BTK	Conservation, disordered region, goodness-of-fit, binding site, solvent accessibility
GJB1	Residue number, on modelled regions, conservation, disordered region
F9	Residue number, conservation, disordered region, change in residue volume, predicted to impact protein stability, predicted to impact protein-protein interaction
HPRT1	Reference amino acid, alternative amino acid, disordered region, solvent accessibility, goodness-of-fit, predicted to impact protein stability

Table S8. The performance of ProSper in classifying variants of 21 X-linked genes as evaluated using the area under the curve (AUC) of the Receiver Operating Characteristic (ROC), the AUC of the Precision Recall (PR), and the Matthews Correlation Coefficient (MCC) test.

Genes	AUC ROC	AUC PR	MCC
G6PD	0.78	0.75	0.55
ALAS2	0.90	0.90	0.62
RS1	0.88	0.87	0.65
OTC	0.89	0.87	0.67
L1CAM	0.88	0.88	0.69
MTM1	0.89	0.88	0.70
ABCD1	0.92	0.91	0.72
PHEX	0.91	0.90	0.74
CLCN5	0.91	0.91	0.75
F8	0.93	0.93	0.75
GLA	0.95	0.94	0.75
IL2RG	0.91	0.90	0.77
AVPR2	0.92	0.91	0.77
IDS	0.93	0.92	0.77
NDP	0.96	0.95	0.78
PDHA1	0.95	0.95	0.79
OCRL	0.95	0.93	0.83
BTK	0.96	0.96	0.83
GJB1	0.95	0.93	0.84
F9	0.97	0.97	0.85
HPRT1	1	1	1

Table S9a. The gene- or protein-specific pathogenicity thresholds identified in 21 genes for three prediction tools using all of the datasets. The gene-specific threshold was identified using 80% of the predictions from VEST4, REVEL, and ClinPred through repeated (*n*=10) 5-fold cross-validation with random subsampling. The rest (20%) of the predictions from each tool was used to test the newly identified threshold using the Matthews Correlation Coefficient (MCC) test, *i.e.* optimised MCC. The VEST4 predictions were unavailable for *ALAS2* and *NDP* variants in the respective transcripts of interest.

Genes	VEST4 threshold	REVEL threshold	ClinPred threshold
G6PD	0.44±0.02	0.59±0.02	0.53±0.19
ALAS2	-	0.61±0.06	0.74±0.25
RS1	0.70±0.07	0.65±0.00	0.76±0.04
MTM1	0.70±0.07	0.80±0.00	0.95±0.00
OTC	0.59±0.05	0.69±0.07	0.65±0.11
PHEX	0.84±0.05	0.81±0.04	0.89±0.05
F8	0.30±0.00	0.49±0.02	0.59±0.03
IL2RG	0.25±0.00	0.53±0.03	0.40±0.13
L1CAM	0.60±0.05	0.59±0.06	0.95±0.00
CLCN5	0.83±0.04	0.86±0.04	0.95±0.02
IDS	0.43±0.02	0.73±0.02	0.95±0.00
GLA	0.42±0.05	0.36±0.10	0.40±0.02
ABCD1	0.59±0.05	0.71±0.06	0.78±0.03
F9	0.24±0.03	0.45±0.00	0.39±0.09
GJB1	0.29±0.12	0.41±0.03	0.47±0.15
AVPR2	0.64±0.06	0.29±0.05	0.73±0.07
PDHA1	0.62±0.03	0.70±0.00	0.70±0.17
BTK	0.49±0.06	0.37±0.02	0.70±0.00
OCRL	0.70±0.02	0.76±0.15	0.95±0.00
NDP	-	0.50±0.06	0.54±0.13
HPRT1	0.40±0.00	0.52±0.04	0.59±0.03

Table S9b. A comparison of the Matthews Correlation Coefficient (MCC) scores with the optimized MCC for the performance of REVEL, VEST4, and ClinPred using all of the datasets. The optimized MCC was generated using gene- or protein-specific pathogenicity thresholds. The gene-specific threshold was identified using 80% of the predictions from VEST4, REVEL, and ClinPred through repeated (*n*=10) 5-fold cross-validation with random subsampling. The optimized MCC score was generated using the rest (20%) of the predictions from each tool at the threshold identified for each gene. The original MCC score was generated using the suggested and widely used threshold of 0.5. The improvement in prediction performance, i.e. a higher optimized MCC score compared to the original, is highlighted in bold. The VEST4 predictions were unavailable for *ALAS2* and *NDP* variants in the respective transcripts of interest.

	VEST4		REVEL		ClinPred	
Genes	Original	Optimised	Original	Optimised	Original	Optimised
G6PD	0.59	**0.63**±0.12	0.61	**0.63**±0.16	0.52	0.47±0.13
ALAS2	-	-	0.63	0.63±0.15	0.73	0.70±0.10
RS1	0.69	**0.71**±0.08	0.59	**0.64**±0.15	0.69	**0.70**±0.13
MTM1	0.71	**0.75**±0.06	0.58	**0.77**±0.09	0.66	**0.86**±0.07
OTC	0.61	0.60±0.10	0.46	0.44±0.11	0.65	0.63±0.09
PHEX	0.62	**0.74**±0.08	0.58	**0.66**±0.07	0.62	**0.72**±0.07
F8	0.75	**0.79**±0.02	0.82	0.80±0.03	0.74	**0.76**±0.02
IL2RG	0.74	0.73±0.07	0.78	0.71±0.10	0.78	0.69±0.09
L1CAM	0.73	**0.79**±0.08	0.65	0.61±0.09	0.58	**0.75**±0.06
CLCN5	0.59	**0.70**±0.09	0.42	**0.64**±0.09	0.58	**0.81**±0.07
IDS	0.67	0.66±0.07	0.64	**0.75**±0.06	0.79	**0.89**±0.05
GLA	0.57	0.53±0.14	0.53	0.47±0.14	0.52	**0.54**±0.11
ABCD1	0.72	0.68±0.04	0.68	**0.71**±0.05	0.75	**0.79**±0.04
F9	0.39	**0.40**±0.12	0.57	**0.59**±0.09	0.50	0.38±0.11
GJB1	0.49	**0.51**±0.13	0.67	**0.68**±0.09	0.53	0.40±0.17
AVPR2	0.76	0.73±0.08	0.62	**0.68**±0.05	0.73	**0.74**±0.06
PDHA1	0.70	0.70±0.09	0.60	**0.77**±0.11	0.80	0.77±0.06
BTK	0.80	0.72±0.16	0.80	0.74±0.10	0.72	**0.77**±0.13
OCRL	0.78	**0.92**±0.06	0.76	0.70±0.10	0.62	**0.89**±0.07
NDP	-	-	0.63	0.58±0.12	0.75	0.69±0.12
HPRT1	0.73	0.44±0.33	0.62	0.40±0.42	0.79	0.44±0.45

Table S10a. The gene- or protein-specific pathogenicity thresholds identified in 21 genes for three prediction tools using a subset of each of the datasets. For each gene, the dataset was balanced using undersampling, *i.e.* using a random subset from the majority class to match the number of variants in the minority class. The gene-specific threshold was identified using 80% of the predictions from VEST4, REVEL, and ClinPred through repeated (*n*=10) 5-fold cross-validation with random subsampling. The rest (20%) of the predictions from each tool was used to test the newly identified threshold using the Matthews Correlation Coefficient (MCC) test, *i.e.* optimised MCC. The VEST4 predictions were unavailable for *ALAS2* and *NDP* variants in the respective transcripts of interest.

Genes	VEST4 threshold	REVEL threshold	ClinPred threshold
G6PD	0.49±0.09	0.72±0.08	0.74±0.14
ALAS2	-	0.75±0.11	0.89±0.04
RS1	0.82±0.03	0.74±0.07	0.86±0.09
MTM1	0.69±0.07	0.82±0.02	0.95±0.00
OTC	0.73±0.11	0.78±0.05	0.87±0.03
PHEX	0.85±0.00	0.79±0.05	0.92±0.05
F8	0.45±0.05	0.58±0.06	0.78±0.09
IL2RG	0.40±0.06	0.55±0.00	0.75±0.17
L1CAM	0.56±0.02	0.40±0.15	0.95±0.00
CLCN5	0.82±0.05	0.87±0.04	0.94±0.03
IDS	0.69±0.10	0.77±0.02	0.95±0.02
GLA	0.68±0.10	0.59±0.10	0.80±0.13
ABCD1	0.70±0.09	0.79±0.05	0.94±0.05
F9	0.50±0.00	0.63±0.12	0.73±0.18
GJB1	0.49±0.08	0.59±0.08	0.72±0.14
AVPR2	0.69±0.03	0.42±0.07	0.85±0.08
PDHA1	0.68±0.08	0.74±0.05	0.92±0.11
BTK	0.67±0.05	0.72±0.04	0.92±0.08
OCRL	0.70±0.02	0.66±0.15	0.95±0.00
NDP	-	0.59±0.06	0.59±0.02
HPRT1	0.58±0.18	0.79±0.07	0.79±0.12

Table S10b. A comparison of the Matthews Correlation Coefficient (MCC) scores with the optimized MCC scores for the performance of REVEL, VEST4, and ClinPred using a subset of each dataset. For each gene, the dataset was balanced using undersampling, *i.e.* using a random subset from the majority class to match the number of variants in the minority class. The optimized MCC was generated using gene- or protein-specific pathogenicity thresholds. The gene-specific threshold was identified using 80% of the predictions from VEST4, REVEL, and ClinPred through repeated (*n*=10) 5-fold cross-validation with random subsampling. The optimized MCC score was generated using the rest (20%) of the predictions from each tool at the threshold identified for each gene. The original MCC score was generated using the suggested and widely used threshold of 0.5. The improvement in prediction performance, i.e. a higher optimized MCC score compared to the original, is highlighted in bold. The VEST4 predictions were unavailable for *ALAS2* and *NDP* variants in the respective transcripts of interest.

	VEST4		REVEL		ClinPred	
Genes	Original	Optimised	Original	Optimised	Original	Optimised
G6PD	0.59	0.58±0.14	0.61	0.51±0.19	0.52	0.50±0.14
ALAS2	-	-	0.63	0.63±0.20	0.73	**0.76**±0.14
RS1	0.69	0.61±0.15	0.59	**0.61**±0.10	0.69	**0.72**±0.14
MTM1	0.71	0.71±0.09	0.58	**0.72**±0.09	0.66	**0.86**±0.06
OTC	0.61	0.61±0.12	0.46	**0.60**±0.12	0.65	**0.77**±0.08
PHEX	0.62	**0.77**±0.08	0.58	**0.67**±0.07	0.62	**0.75**±0.08
F8	0.75	**0.86**±0.04	0.82	**0.83**±0.02	0.74	**0.83**±0.03
IL2RG	0.74	0.66±0.12	0.78	0.70±0.13	0.78	0.68±0.12
L1CAM	0.73	**0.82**±0.05	0.65	0.60±0.11	0.58	**0.78**±0.07
CLCN5	0.59	**0.66**±0.08	0.42	**0.66**±0.08	0.58	**0.75**±0.07
IDS	0.67	**0.71**±0.04	0.64	**0.77**±0.08	0.79	**0.89**±0.08
GLA	0.57	**0.74**±0.11	0.53	**0.61**±0.16	0.52	**0.75**±0.12
ABCD1	0.72	0.72±0.06	0.68	**0.76**±0.06	0.75	**0.88**±0.07
F9	0.39	**0.74**±0.13	0.57	**0.60**±0.17	0.50	**0.68**±0.14
GJB1	0.49	**0.84**±0.13	0.67	0.66±0.15	0.53	**0.67**±0.21
AVPR2	0.76	**0.77**±0.07	0.62	**0.66**±0.13	0.73	**0.77**±0.08
PDHA1	0.70	0.60±0.17	0.60	**0.67**±0.17	0.80	0.78±0.05
BTK	0.80	**0.88**±0.08	0.80	**0.85**±0.09	0.72	**0.82**±0.12
OCRL	0.78	**0.92**±0.04	0.76	0.74±0.11	0.62	**0.88**±0.05
NDP	-	-	0.63	0.62±0.28	0.75	**0.88**±0.15
HPRT1	0.73	0.59±0.44	0.62	0.61±0.38	0.79	0.76±0.40
